# Systems biology approach to studying proliferation-dependent prognostic subnetworks in breast cancer

**DOI:** 10.1038/srep12981

**Published:** 2015-08-10

**Authors:** Qianqian Song, Hongyan Wang, Jiguang Bao, Ashok K. Pullikuth, King C. Li, Lance D. Miller, Xiaobo Zhou

**Affiliations:** 1 Division of Radiology, Wake Forest School of Medicine, Winston-Salem, NC 27157, USA.; 2 School of Mathematical Sciences, Beijing Normal University, Beijing, 100875, P R China.; 3 Department of Cancer Biology, Wake Forest School of Medicine, Winston-Salem, NC, 27157, USA.

## Abstract

Tumor proliferative capacity is a major biological correlate of breast tumor metastatic potential. In this paper, we developed a systems approach to investigate associations among gene expression patterns, representative protein-protein interactions, and the potential for clinical metastases, to uncover novel survival-related subnetwork signatures as a function of tumor proliferative potential. Based on the statistical associations between gene expression patterns and patient outcomes, we identified three groups of survival prognostic subnetwork signatures (SPNs) corresponding to three proliferation levels. We discovered 8 SPNs in the high proliferation group, 8 SPNs in the intermediate proliferation group, and 6 SPNs in the low proliferation group. We observed little overlap of SPNs between the three proliferation groups. The enrichment analysis revealed that most SPNs were enriched in distinct signaling pathways and biological processes. The SPNs were validated on other cohorts of patients, and delivered high accuracy in the classification of metastatic vs non-metastatic breast tumors. Our findings indicate that certain biological networks underlying breast cancer metastasis differ in a proliferation-dependent manner. These networks, in combination, may form the basis of highly accurate prognostic classification models and may have clinical utility in guiding therapeutic options for patients.

Breast cancer was the most commonly diagnosed type of cancer among women in the United States in 2012, accounting for 29% of all new cancer cases in women^1^. It is estimated that in 2015 in the U.S. alone, 231,840 new women patients will be diagnosed with breast cancer and an estimated 40,290 deaths may result from morbidity associated with this malignacy^2^. Breast cancer, like other solid tumor types, can metastasize to distant organ sites following surgical and systemic treatment^3^ that is the leading cause of patient mortality. Treatment options including surgery, adjuvant chemotherapy and molecularly targeted therapies may delay or prevent metastasis for some patients, but not others. Breast cancer is characterized by vast heterogeneity at the pathological, clinical and intrinsic molecular levels that may influence treatment options and patient outcomes. These heterogeneities underscore the need for a better understanding of the pathobiological mechanisms associated with breast tumor progression and recurrence that could lead to novel treatment strategies.

Many studies have reported gene-based markers as biological signatures to predict patient outcomes. Wang *et al*.^4^ studied tumor gene expression profiles using average linkage hierarchical clustering. They used two supervised class prediction approaches to identify genes that discriminated between patients who developed distant metastases from those who remained metastasis-free for 5 years. van’t Veer *et al*.^5^ applied supervised classification to identify a gene expression signature that predicted rapid breast cancer recurrence, and provided a strategy to select patients that would benefit from adjuvant therapy. In both studies, the prognostic genes appeared to represent a number of known signaling pathways related to cancer, but the individual contributions of each to tumor metastatic potential was uncertain.

A number of studies have reported on prognostic markers related to cell proliferation and immunity. Sotiriou *et al*.^6^ identified 97 genes associated with histologic grade and functionally involved in cell proliferation. Wirapati, *et al*.^7^ compared the prognostic performances of nine gene signatures; their prognostic abilities largely depended on their inclusion of proliferation-associated genes. In contrast, in a study by Rody *et al*.^8^, seven surrogate markers of tumor-infiltrating immune cells were identified from over 600 genes functionally associated with leukocyte biology. Although some studies have identified gene markers as principally biology-driven predictors of breast cancer outcomes, their results were rarely consistent^9^,^10^. Most published gene markers have had limited or negligible clinical utility to predict individual patient outcomes^11^,^12^. In response to these issues, methods based on the concept of network markers were developed to provide more meaningful predictive information than traditional gene-focused methods^11^,^13^. For example, signaling nodes and axes responsible for interconnecting many differentially expressed genes are not detected through conventional differential expression analysis. Compared with individual gene markers without network information, such subnetwork markers achieved higher predictive accuracies. In the work of Chuang *et al*. prognostic subnetworks were identified and validated as more accurate predictors of the relative risk for disease progression than established gene markers^13^.

Thus, here we propose a method for discovering survival-associated prognostic subnetworks that would uncover biological mechanisms in metastatic breast cancer, which are either dependent or independent of tumor proliferative capacity. We utilized a previously assembled breast tumor microarray meta-cohort of 1,954 tumor expression profiles annotated for clinical outcomes^14^. Based on the prevalence of proliferation genes in breast tumor expression profiles^14^,^15^, we stratified the metacohort for tumor proliferative capacity using our previously defined proliferation (P) metagene^14^. We partitioned all breast cancer cases into three proliferation subgroups (P-high, P-inter and P-low) based on the P metagene score (i.e. the average expression level of the genes comprising the metagene). Then we applied an approach integrating the greedy search algorithm with the Cox proportional-hazards model to discover subnetworks associated with the distant metastasis-free survival time (DMFS) in different proliferation groups.

## Results

### Subnetwork signatures characteristic of proliferation tertiles

In spite of the various studies that have identified gene signatures that can predict and distinguish patient responses according to clinical responses and outcomes, a clear consensus among these gene signatures has been elusive. We hypothesized that in addition to gene signatures, an underlying biological protein-protein interaction information could be uncovered that will bolster the predictive power when used in conjunction with gene signatures. To this end, we stratified the patient samples into tertiles of Proliferation-high, -intermediate and –low (P-high, P-inter and P-low respectively) according to their proliferative capacity (See Methods and Nagalla *et al*.^14^).

With the P-high group as an example, we performed a 10-fold cross-validation for 1000 iterations to obtain robust assignments of patients (good outcome = 225, poor outcome = 427, Figure S1). The log-rank P-value of such classification of patients was 2.62 × 10^−04^, which illustrates the robustness of our approach. Through our approach, the survival prognostic subnetworks (SPNs) were obtained by the three tests filtering the primary subnetworks (PNs, see Methods C).

### SPNs stratify patients into different risk sub-groups

#### P-high group

**Figure 1:**
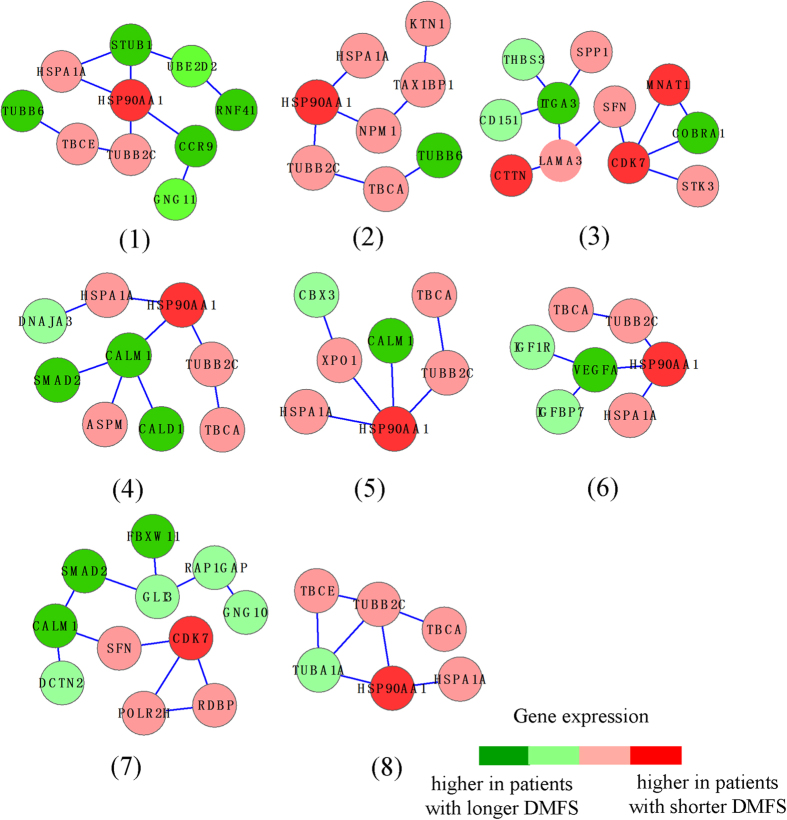
SPNs discovered in the P-high group, i.e. P-high SPNs. There are 8 SPNs discovered in the high proliferation (P-high) group. P-high SPNs contain important genes, like the HSP90AA1, CALM1, VEGFA, etc. Genes are color coded (i.e. red/green: genes that are overexpressed/underexpressed in patients with shorter DMFS). In the color bar, deep green means that log2(fold change) <0, ligh green or light red represents that 0< fold change <1, while deep red means that log2(fold change) >0. Details can be found in Results.

**Figure 2:**
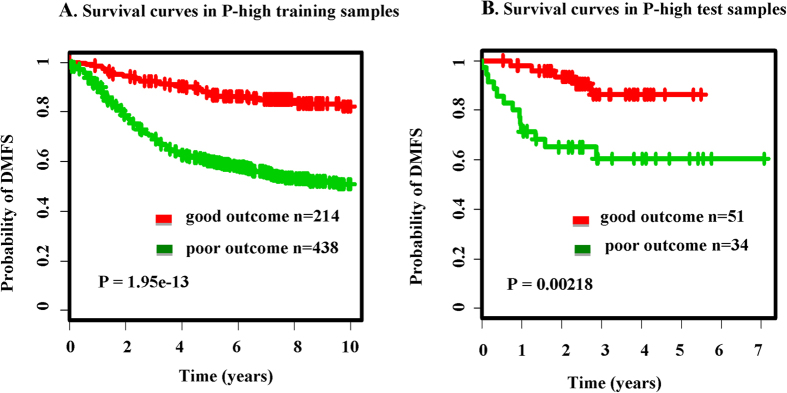
Survival analysis of P-high subsets in training set and test set. (**A**) the survival curves by Kaplan-Meier analysis in training set (P-high subset of our breast cancer dataset). (**B**) the survival curves in test set (P-high subset of GSE25055 dataset). Red color represented patients that were classified as good outcome, and green color represented patients classified as poor outcome.

By applying our approach to the P-high tertile of the training set, we identified 8 SPNs, which we call P-high SPNs (Fig. 1). The genes in P-high SPNs are colored in red or green to represent over-expression or under-expression, respectively, in patients with shorter DMFS. The adjusted P-value (P-value of chi-square test for Cox model corrected using Bonferroni adjustments) of the SPNs are shown in Supplementary Table S1. In the training set (P-high subset, n = 652), patients were classified by the 8 SPNs into two subgroups. Kaplan-Meier estimator revealed significant difference between the two subgroups of patients (Fig. 2, log-rank *p* value = 1.95 × 10^−13^). 214 patients with longer DMFS in one subgroup were classified as good-outcome (red color), while the other 438 patients with shorter DMFS in the other subgroup were classified as poor-outcome (green color) (Fig. 2A). In the test set (P-high subset, n = 85), patients were stratified by the 8 SPNs into two subgroups (Fig. 2B). The difference between the two subgroups was significant (log-rank *p* value = 0.00218). 51 patients were classfied as good outcome and 34 pateints were classifed as poor outcome. The accuracy of stratification in test set was 88.24%, indicative of higher predictive power of P-high SPNs. We found that the size ratios of the subgroups with different outcome varied between training and test sets. For the P-high group, the size ratio was 214/438 (good vs poor outcome) in training subgroup, whearas it was 51/34 (good vs poor outcome) in test subgroup. By comparing the distribution of patients’ DMFS in the two datasets, we observed that they have different proportion of longer/shorter DMFS.

#### P-intermediate and P-low groups

We identified 8 SPNs and 6 SPNs respectively in P-inter and P-low tertile of training set, which are called P-inter SPNs and P-low SPNs. The P-inter and P-low SPNs are shown in Figure S2 and S3. The genes in P-inter and P-low SPNs colored with red or green represented over-expression or under-expression, repectively, in patients with shorter DMFS.

**Figure 3:**
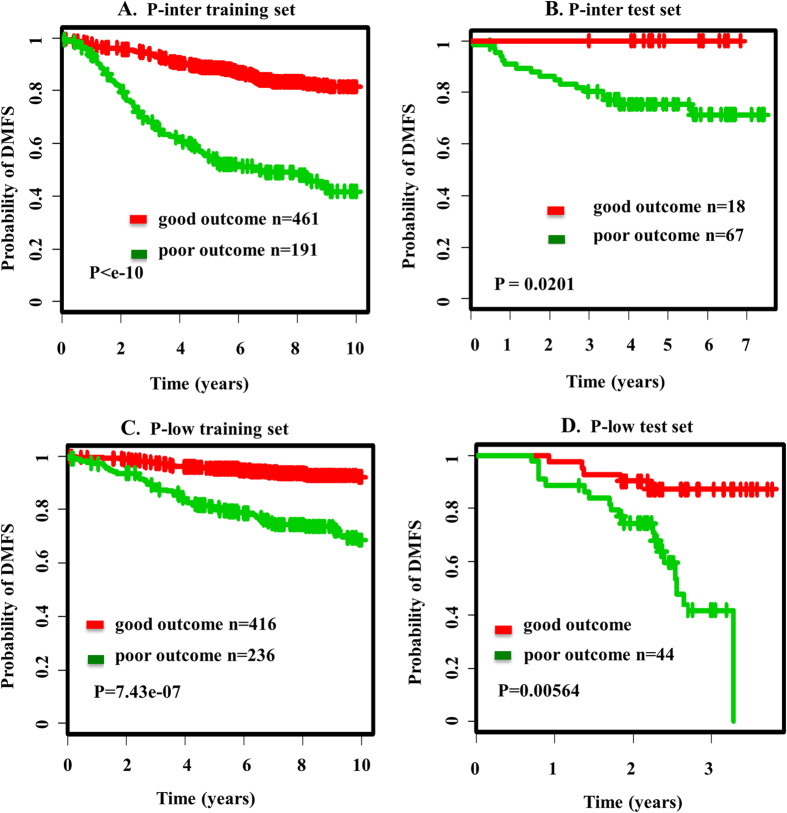
Survival analysis of P-inter/P-low subsets in training set and test set. (**A**) the survival curves by Kaplan-Meier analysis in the P-inter training set. (**B**) the survival curves by Kaplan-Meier analysis in the P-inter test set. (**C**) the survival curves by Kaplan-Meier analysis in the P-low training set. (**D**) the survival curves by Kaplan-Meier analysis in the P-low test set.

In P-inter tertile (n = 652) of training set, 461 patients were classifed as good-outome, while the other 191 patients were classifed as poor-outcome (Fig. 3A). In P-low tertile (n = 652) of training set, 416 patients were classifed as good-outome, while the other 236 patients were classifed as poor-outcome (Fig. 3C).

In the P-inter and P-low tertile of test sets, we utilized P-inter and P-low SPNs for patient stratification based on clinical outcome. In P-inter tertile (n = 85) of test set, 8 patients were classifed as good-outome, while the other 67 patients were classifed as poor-outcome (Fig. 3B). In P-low tertile (n = 85) of test set, 41 patients were classifed as good-outome, while the other 44 patients were classifed as poor-outcome (Fig. 3D). Survival curves (with significant log-rank P-values) of P-inter and P-low tertile of training set are shown in Fig 3. The significant P-value (for P-inter, log-rank *p* = 0.0201; for P-low, log-rank *p* = 0.0056) in test set showed the stratification power of P-inter and P-low SPNs. The classification accuracy is shown in Figure S4. In both training and test set, our SPNs has the potential to deliver high accuracy.

Taken together, P-high/P-inter/P-low SPNs are robust in patients statification and DMFS prediction. These results further indicate that our approach can effectively and accurately stratify the breast cancer patients.

### Enrichment analysis of SPNs

#### Biological Process and KEGG Pathway Enrichment

We examined the SPNs for their potential enrichment in biological process (BP) sets (825 in MSigDB database) and pathways (186 in MSigDB database). Enrichment of a certain biological process or pathway is indicated by yellow, whereas non-enrichment is indicated by blue.

**Figure 4:**
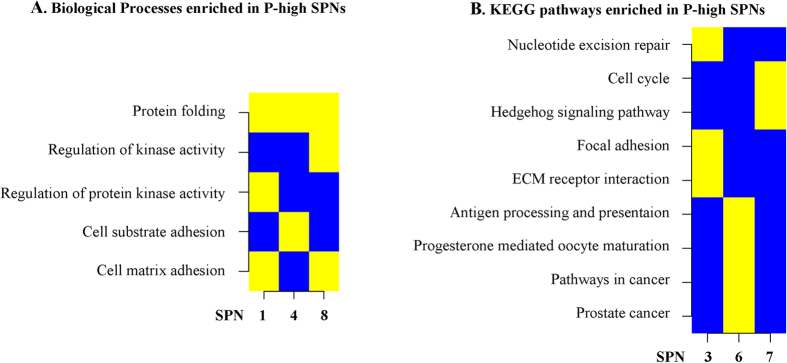
Enrichment analysis of P-high SPNs in BP sets and KEGG pathway sets. (**A**) visualize the enriched categories of Biological Process (BP) sets. (**B**) visualize the enriched categories of KEGG pathway sets. Enriched biological process or pathway (i.e. enrichment) was indicated by yellow, whereas non-enrichment was indicated by blue.

For the P-high SPNs, some biological processes, like the protein folding process, were enriched in 3 SPNs (i.e. SPN 1, 4 and 8). Nine KEGG pathways were enriched in 3 SPNs (i.e. SPN 3, 6, 7) (Fig. 4A,B). Of these pathways, SPN 3 enriched in the ECM receptor interaction pathway and the antigen processing and presentation pathway, which are implicated in tumor progression and breast cancer metastasis^16^,^17^. Interestingly, SPN 6 enriched in prostate cancer pathway and general pathways in cancer. The cell cycle pathway was enriched in SPN 7.

With regard to P-inter SPNs, 14 biological processes including mainly the metabolic, catabolic process and ubiquitin cycle process were separately enriched in SPN 2 and SPN 4 (Figure S5. A). While SPN 2, 5, 6 enriched in the Ubiquitin mediated pathway (Figure S5. B), which has a role in transcription regulation^18^ and also links to cytoskeletal dynamics, cell adhesion and migration^19^. Interestingly, some SPNs enriched in apoptosis pathway, pathway in cancer, and the colorectal cancer pathway.

With respect to P-low SPNs, 15 biological processes including the negative regulation of cell cycle and cell proliferation were respectively enriched in SPN 2 and SPN 5 (Figure S6. A). Five P-low SPNs (i.e. SPN 1, 2, 4, 5, 6) enriched in several signaling pathways, including the MAPK, ERBB, chemokine, NK cell-, T-cell-, B-cell, transendothelial migration signaling pathways, which may collectively have functions in cell proliferation, metastasis and survival^20^ and may play roles in the immune system^21^ function as well.

The pathways that were enriched in P-inter and P-low SPNs were largely distinct. On the one hand, several cancer associated pathways were enriched in both P-inter and P-low SPNs, whereas the types of cancer differed between these two categories. On the other hand, several catabolic processes and protein degradation pathways were enriched in P-inter SPNs, whereas P-low SPNs enriched in immune function pathways (especially P-low SPN 2) and cell cycle regulation.

### Patient stratification across treatment regimens: clinical and intrinsic subtypes

Given the classification ability of SPNs in patients regardless of their treatment and other clinical characteristics, we evaluated the SPNs’ classification performance in certain therapeutic subpopulations of test set. Detailed clinical and pathological characteristics of test set is provided in the Supplementary Table S2.

**Figure 5:**
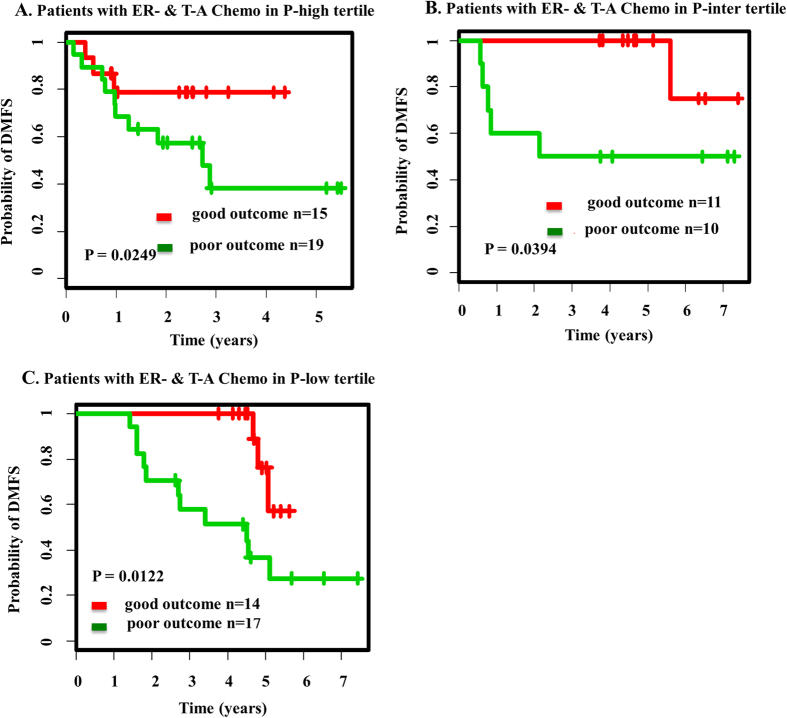
Survival analysis of patients across treatment regimens. (**A**) the classification of subpopulations with ER-negative subtype that received taxane & anthracycline chemotherapy (T-A Chemo) in P-high test set. (**B**) the survival curves of estrogen receptor-negative (ER-) patients who received taxane & anthracycline chemotherapy (T-A Chemo) in P-inter test set. (**C**) the survival curves of estrogen receptor-negative (ER-) patients who received taxane &anthracycline chemotherapy (T-A Chemo) in P-low test set.

We focused on classifying subpopulations with ER-negative subtype that received taxane and anthracycline chemotherapy in P-high, P-inter and P-low test set. Figure 5 shows the survival curves of these three subpopulations. In the estrogen receptor-negative (ER-) group in P-high test set, patients who received taxane and anthracycline chemotherapy, 15 and 19 patients were classified good- and poor- outcome (log-rank *p* = 0.0249) respectively. In P-inter test set, patients with ER- that received taxane and anthracycline chemotherapy were statistically significantly classifed as 11 good outome and 10 poor outcome (log-rank *p* = 0.0394). The subpopulations in the P-low test set were classified as 14 good and 17 poor outcome patients (log-rank *p* = 0.0122). Therefore subpopulations with certain clinical subtype were also significantly segregated by P-high/P-inter/P-low SPNs (Fig. 5) lending support for SPNs’ potential for prognostics across treatment regiments and clinical subtype.

### Comparison with other algorithms

We compared our method with that of Cox-based Ridge regression^22^,^23^ and the CRANE method^24^. Since our method was based upon the proliferation capacity of breast tumor, we employed the P-high tertile of training and test set to perform these comparisons.

#### Cox-based Ridge Regression method

**Figure 6:**
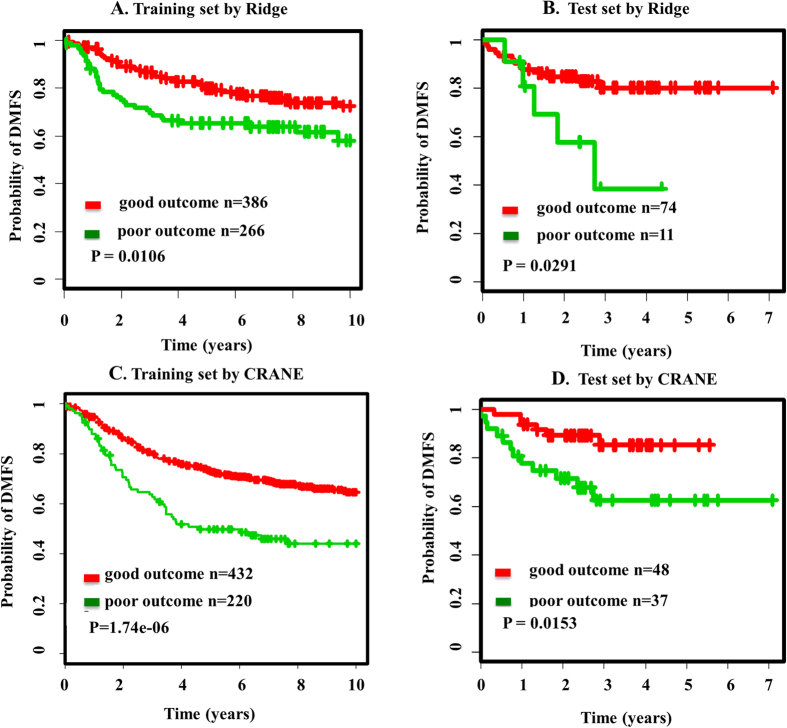
Survival analysis based on signatures discovered by other algorithms. (**A**) the survival curves of training set based on gene markers discovered by the Cox-based ridge regression model. (**B**) the survival curves of test set based on gene markers discovered by the Cox-based ridge regression model. (**C**) the survival curves of training set based on network markers discovered by the CRANE method. (**D**) the survival curves of test set based on network markers discovered by the CRANE method.

We trained the Cox-based Ridge regression model on the training set (P-high, n = 652), and selected top gene signatures. Then we assessed the significance of these gene signatures on test set (P-high, n = 85). The survival curves and corresponding statistical significance are shown in Figure 6. The log-rank P-values on training and test sets are 0.0106 and 0.0291, respectively (Fig. 6A,B). The classification accuracy is 57.06% (Figure S4), which is lower than the classfication accuracy based on our SPNs.

#### The CRANE method

We next applied the CRANE method on the training set to identify subnetwork signatures, and then to validate signature in test set. Here, we binarized gene expression profiles on both sets to implement CRANE.

We normalized the gene expression profiles, and set the top fraction (25%) of the gene expression matrix to “High expression” and the rest to “Low expression”. With the bottom-up searching algorithm on the training set, we identified 11 subnetwork signatures. We used these 11 subnetworks, to classify the samples in both training and test set (Fig. 6C,D). The corresponding statistical significance and accuracy (together with precision and recall) are presented in Figure 6 and Figure S4 (for test set: P-value = 0.015, accuracy is 74.71%).

#### Our method

As seen in Figs 2 and 6 and Figure S4, our method outperformed the other two methods in predicting metastasis of breast cancer patients. In test set, our method has the potential to deliver higher significance and accuracy (P-value = 0.00281, accuracy is 88.24% for test set). Comparison the P-values and accuracy of patient stratification of our method with CRANE indicated that our method was more effective than CRANE.

### Comparison with other signatures

In order to test the performance of our SPNs with previously identified gene signatures that have been shown to have predictive power, we compared our SPNs with that of Wang *et al*.’s 76-gene signatures^4^. We applied our SPNs to the dataset used to obtain Wang *et al*.’s 76-gene signatures (GEO Accession No: GSE2034). Since our SPNs are proliferation dependent, the GSE2034 dataset was divided into three proliferation subsets by the nearest shrunken centroid classifier^25^ as described in the Methods section. After assigning the GSE2034 dataset to three proliferation subsets, we applied our corresponding SPNs to the three proliferation subsets. The survival curves of the three proliferation subsets are presented in Figure S7. As shown in Figure S7, the significant P-values suggest that our SPNs indeed have strong predictive power, and have comparable performance to that of Wang *et al*.’s 70-gene signatures. Besides, it also suggests that the proliferation index is very important in predicting breast cancer patients’ metastasis.

Additionally, the Chuang *et al*.’s study^11^ identified 149 and 243 discriminative subnetworks based on van de Vijver *et al*. (2002)^26^ and Wang *et al*. (2005)^4^ data sets. A compendium including all of their subnetworks is available online via the Cell Circuits database^27^. We compared the sub-networks identified in our study with those identified by Chuang *et al*.^11^. Interestingly, there were no completely overlap between our SPNs with their discriminative subnetworks. One possible explanation for the different subnetwork signatures is that, our study focuses on discovering subnetwork signatures dependent on proliferation capacity, while Chuang *et al*.’s study focuses on primary breast cancer patients. Besides, the datasets used in Chuang *et al*.’s and our studies are different in terms of patients’ subtypes: the Chuang *et al*.’s dataset contain mostly lymph-node-negative primary breast cancer patients, whereas our dataset includes all of the six intrinsic molecular subtypes. Furthermore, the protein-protein interaction databases used as the searching space are also different in the two studies.

It is interesting to note that there are some common enriched biological processes in the discovered network signatures obtained by both studies, e.g. the process of cell proliferation and apoptosis, metabolic process, etc. In addition, we identified 27 common genes between our SPNs and Chuang’s network signatures (orange color in the first column “Gene Symbol” of Supplementary Table S4).

In addition, we summarized the genes for our SPNs, genes identified by the Cox-based Ridge regression, and genes in the subnetworks identified by CRANE. Supplementary Table S4 shows the overlap and unique genes between different signatures. In the grid, the column and row of red cell represent the network signature and its corresponding gene, respectively. Supplementary Table S4 indicates that the signatures obtained from our method share 10 genes with the CRANE, 5 genes with the Cox-based Ridge regression method, whereas the CRANE and the Cox-based Ridge regression method shared only 2 genes.

## Discussion

In this study, the proliferation metagene was applied to divide the samples. Patients were clustered to proliferation groups based on the expression values of proliferation metagene through the hierarchical clustering. In each proliferation group, patients were stratified based on our SPNs, and the survival analysis was conducted by the Kaplan-Meier Method. According to the previous study^14^ and our approach, the proliferation metagene performed well when patients were divided into three groups. However, the statistical power diminishes when patients are divided either into two or four groups (Figure S8 and S9 respectively). Thus we utilized the three proliferative groups (P-high, P-inter and P-low) to identify SPNs.

In our study, we assumed that all the genes in the sub-networks have the same prediction value for DMFS. It may be important to consider that different genes in the sub-networks, especially the core genes might differ in their predictive value. For example weighting HSP90AA1 in sub-network 1, 2, 5, 6 from the P-high tertile may give a better prediction for DMFS. Here we took the frequency of the genes appeared in the SPNs as a weight for those gene. Figure S10 presents the P-values in P-high (p = 0.145) and P-inter (p = 0.508) group that are non-significant, while in the P-low group, the P-value (p = 0.00528) is more significant. Thus assigning weights to core genes doesn’t always provide a better prediction for the DMFS. However, the mean value is able to capture the joint role of genes in a subnetwork. We obtained significant and reproducible subnetwork signatures based on mean value of z-scores consistent with previous studies^13^,^14^. Thus the mean value of normalized gene expression data is an efficient and effective metric to characterize the activity of a subnetwork.

To address if these SPNs from certain P tertile have a general impact on DMFS, we applied the SPNs identified in one P-tertile to test in the other P tertiles (Figure S11). By comparing the survival curves for each tertile using corresponding SPNs, we observed that the specific SPNs have great impact on its corresponding P-tertile, but much less impact on the other P-tertiles, which demonstrates the SPNs depends closely on the proliferation tertile.

Next we tested the hypothesis that gene signatures when used concomittantly with protein interaction data reflective of related signaling and proteomic landscape can provide robust prognostic tools in breast cancer. The SPNs we identified within each proliferation tertile are largely exclusive to the specific tertile, however, while a few individual genes overlap between proliferation tertiles (e.g. RAC1 and RAP1B between P-inter and P-low SPNs), the majority of interacting genes (that interact with the overlapping genes in SPN assignment) remain unique to a specific proliferation tertile. Besides, our SPNs have the potential to deliver higher significance and accuracy than other signatures in stratifying patients.

In the P-high tertile, most subnetworks contained HSPA1A, HSP90AA1 and TUBB2C (i.e., 6/8 SPNs = 75%) whereas the rest share CDK7 and SFN, (2/8 SPNs = 25%). While the former share a few other genes between their component SPNs, CDK7 and SFN (stratifin) interact with entirely different set of genes in SPN3 and SPN7. HSPA1 is a negative regulator of apoptosis and plays important roles in cell growth and cell proliferation^28^. The related HSP90AA1^29^ codes for stress-inducible heat shock protein 90A. The Class I cytosolic HSP90, assisted by co-chaperones and accessory proteins, aids in the folding of a diverse class of proteins including kinases, G protein-coupled receptors (GPCRs), ion channels, transcription factors, and nuclear hormone receptors. A role in oncogenic signaling has been implicated by HSP90’s ability to bind and stabilize mutated oncogenic proteins^30^. HSP90 also mediates antigen cross-presentation by antigen-presenting cells (APC) to activate CD8 + T-cells^31^. TUBB2C, has been implicated in MHC class I protein binding involved in natural killer cell-mediated cytotoxicity^32^. Furthermore, beta class II tubulin predominates in most breast tissues, and nuclear beta (II) may be a useful marker for detection of tumor cells^33^. A conclusion that can be drawn from HSPA1A, HSP90AA1 and TUBB2c containing SPNs is that by selectively interacting with additional redundant and unique genes, these three genes may influence different signaling pathways in highly proliferative tumors.

In the P-inter group, RAC1 and RACGAP1 (3/8 SPNs = 38%), and SP1(4/8 SPNs = 50%) were present in mutually exclusive subnetworks; the former two present in SPNs1, 3, and 5 while SP1 is excluded from these but present in entirely different subnetworks (i.e., 4, 6, 7, 8). The SP1 (coding for zinc finger transcription factor) containing subnetwork is also enriched for IMPDH2 (inosine-5’-monophosphate dehydogenase 2), HTT (Huntingtin), IL2RB (interleukin 2 receptor β), and IGFBP2 (insulin-like growth factor binding protein 2). RAC1 (a member of the small GTPase family) is the key RAC isoform responsible for regulating ROS generation, actin cytoskeleton and cell adhesion and migration in basic cell biology and during the multiple stages of osteoclast differentiation^34^. RAC signaling has also been implicated in growth factor signaling downstream of EGF and PDGF^35^. The presence of RAC and its negative regulator RACGAP1 in the same SPNs might thus regulate growth factor signaling in P-inter breast tumors. In this regard it is interesting to note that RAC1 is predominantly higher in patients with longer DMFS (green in Figure S2), while its negative regulator RACGAP1 is higher in patients with shorter DMFS. From these data, we could infer that consistently active RAC1 at the protein level due to lack of or low expression of its negative regulator might enhance RAC signaling that might result in shorter DMFS perhaps by promoting migration and metastasis. The presence of SP1 together with IL2 signaling regulators, IGFBP2, IMPDH2 and HTT in separate subnetworks that excludes RAC signaling component might indicate the presence of at least two non-redundant mechanisms in P-inter breast cancers that may determine patient outcomes.

Of the six SPNs identified in P-low tumors, CTTN (cortactin) and RAP1B are expressed in the same 5/6 SPNs (83%), of which CTNNA1 (Cadherin associated protein, Catenin α1) is associated with 4 of those SPNs. It is interesting to note that RAP1B is consistently higher in patients with longer DMFS whereas CTTN and CTNNA1 are consistently highly in patients with shorter DMFS suggesting a potential cross regulation or feedback mechanism between these interacting hubs. As one of the most common genes in P-low SPNs, CTNN can enhance the interaction of tumor cells with endothelial cells and the invasion of tumor cells into bone tissues, which contribute to tumor metastasis^36^. CTTN plays a dual role in: 1) regulating the interactions of adherens-type junctions components^37^ and 2) organizing the cytoskeleton and cell adhesion structures of epithelia and carcinoma cells^38^. CTTN is overexpressed in a varity of tumors including breast, hepatocellular, bladder, head and neck tumors where its aberrant regulation was implicated in tumor cell invasion and metastasis^39^,^40^. Interestingly CTNNA1 is also a critical regulator of actin cytoskeleton and adherens junction and cell-substrate interactions^41^,^42^. Consistent with higher CTNNA1 potentially playing a role in patients with shorter DMFS, in P-low tumors, CTNNA1 was recently demonstrated to be downregulted in highly proliferative basal-like tumors acting through NFkB pathway and correleted to clinical outcomes^43^. Both RAP1A and RAP1B belong to the RAS superfamily; RAP1B is the dominant isoform in B cells, and can regulate B-cell development and T-cell dependent humoral immunity^44^. RAP1 counteracts mitogenic signaling in certain diseases in part by interacting with RasGAPs and Raf competitively and has been implicated in the regulation of vascular barrier function. Thus higher RAP1 expression may reduce oncogenic signaling leading to longer DMFS in P-low patients (Figure S3).

In summary, we have utilized a systems approach to integrate gene expression data and patient survival data with protein interaction networks at discrete windows of tumor proliferative biology. As more comprehensive transcriptomic information becomes available (e.g., miRNA and lncRNA data), we will gain a greater depth of understanding of the mechanisms that underlie such biology-dependent prognostic interactions. By narrowing salient gene sets or their assembled signatures into functional interaction networks, we demonstrated the utility of using multi-platform modalities to predict patient outcomes based on specific networks that stratified patients by their proliferation profiles. Similar methods may be useful in uncovering the basic mechanisms that underlie patient responses and may pave the way to identification of prognostic biomarkers for different molecular subtypes of cancer and responses to clinical therapy. The predominance of genes implicated in signaling, intercellular traffic, and cytoskeletal dynamics in these SPNs suggest that it may be practical to formulate testable hypotheses *in vitro* and *in vivo* based on the approach used here.

## Methods

### Datasets and tools

#### Breast cancer microarray dataset

The dataset consists of 15 different breast cancer microarray datasets (total n = 2,116 cases) with the corresponding clinical annotations that were extracted from public data repositories^45^. Most data analysis were performed in the R environment implemented in Bioconductor. Raw array data (CEL files) were normalized using the justMAS function in MAS5.0, in the *simpleaffy* library in the R software. The specific array platforms employed were the HG-U133A, HG-U133 PLUS 2.0 and HG-U133A2 gene chips. Only probe sets common to all chip types were utilized, which resulted in 22,268 probe sets in all study populations. Cross-population batch effects were corrected using the COMBAT empirical Bayes method^46^. Of the initial 2,116 tumor profiles, 1,954 samples were annotated for distant metastasis-free survival (DMFS) time and recurrence event that were used as training set in our study.

#### Test set

This dataset, referenced by accession number GSE25055^47^,^48^ in the GEO database, contained expression profiles of 59 breast tumor samples with metastasis and 196 breast tumor samples without metastasis. The patient samples in GSE25055 were collected by the prospective multicenter study conducted between June 2000 and March 2010 at the M. D. Anderson Cancer Center^47^. We normalized the data using the robust multi-array average (RMA) expression measure^49^ to adjust for differences of study sources. All 255 patient data were annotated for distant metastasis-free survival time (DMFS). Clinical and pathological characteristics of patients and their tumors in the test set are shown in Supplementary Table S2.

#### Human functional protein interaction database (FI)

This database^50^ was constructed by combining curated interactions from Reactome and other pathway databases (including Reactome^51^, Panther^52^, CellMap, NCI Pathway Interaction Database^53^, KEGG^54^, and TRED database^55^), with uncurated pairwise relationships gleaned from physical PPIs in human and model organisms, protein interactions generated from text mining, and GO annotations. The naïve Bayes classifier (NBC) was applied to distinguish high-likelihood FIs from non-functional pairwise relationships as well as outright false positives to construct the final FI network.

#### Biological process (BP) sets

The biological process sets in MSigDB database of GSEA^56^ used the tree structure of GO terms (i.e., parent/child relationships) to construct the process. The resulting sets in MSigDB were “flat” in the sense that they lacked the topology information such as parent – child relationships among GO terms.

#### Statistical programs for analyses

The R software was adopted to implement our approach. We wrote a R program for the greedy search algorithm to identify the subnetwork signatures (SPNs) in different proliferation group of patients (see below). After SPNs were identified, hierarchical cluster (“hcluster” function in R) was used to stratify the patients. Given the binary stratification of patients, we used the “survival” package to perform survival analysis. We applied the “coxph” function to the Cox proportional hazard model, the “survfit” function to create the Kaplan-Meier survival curves while “survdiff” function was utilized to test the difference between two survival curves.

### Patient stratification according to proliferation tertiles

#### A. Patient stratification by tumor proliferation metagene

**Figure 7:**
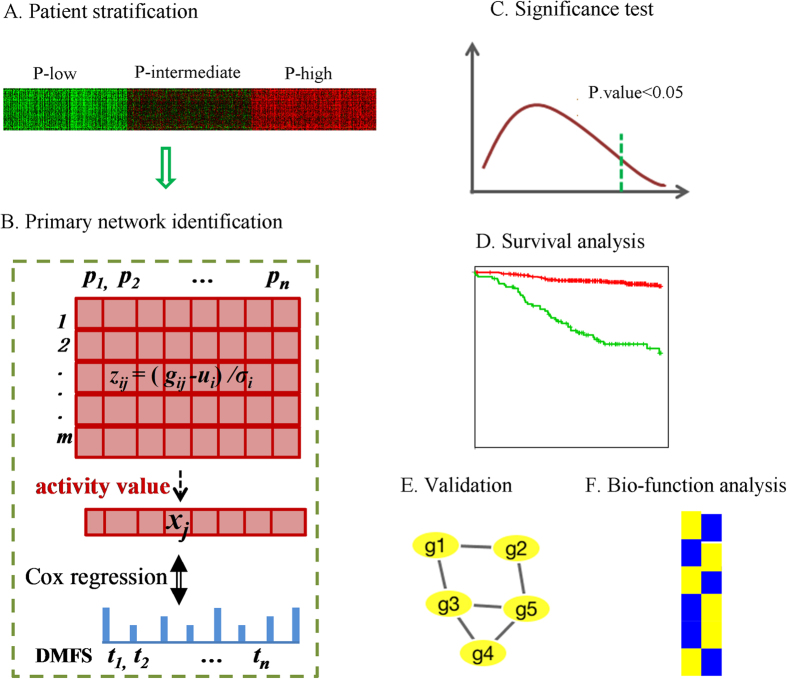
Identification and analysis of the proliferation-dependent survival prognostic network markers (SPNs). (**A**) 1,954 breast cancer patients were divided to three proliferation groups based on the proliferation metagene. (**B**) Assume a subnetwork containing *m* genes. g_ij_ was the gene expression value of the *i-th* gene in *j-th* patient, and *g*_*ij*_ was transformed to *z*_*ij*_ by *z*_*ij*_ = (*g*_*ij*_ − *u*_*i*_)/*σ*_*i*_. The activity value *x*_*j*_ of the *j-th* patient was the average of *z*_*ij*_, i.e. 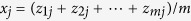. Then the Cox proportional hazard model was used to measure the realtionship between the activity value *x*_*j*_ and *t*_*j*_. Through the P-value of a chi-square test on the Cox model, we defined Score *S* to be *–log(P.value)* as the criteria to select subnetworks. Here a greedy search algorithm was conducted to search the FI network database. At the end of this searching process, we identified primary subnetworks (PNs) with high S scores. (**C**) Three significance tests were conducted to select survival prognostic subnetwork markers (SPNs) from PNs. (**D**) Based on the activity value matrix, the hierarchical clustering was applied to divide patients to two discriminate subgroups. Then Kaplan-Meier analysis was used to study the survival of the two discriminate subgroups. (**E**) Two validation stratigies were used to evaluate our model and SPNs. (**F**) Enrichment analysis of SPNs in biological process (BP) sets and KEGG pathway sets.

In our previous study^14^, we developed the proliferation “metagene”, which includes highly correlated genes with roles in cell cycle and proliferation. Supplementary Table S3 shows the Affymetrix probe sets and corresponding genes that comprise the proliferation metagene. With the proliferation metagene, we clustered the patients through hierarchical clustering. Then the patients was clustered to three gorups with different proliferative capacity, i.e. P-high, P-intermediate and P-low, each containing 652 tumors (Fig. 7A).

#### B. Primary network identification

In each proliferation group, we conducted the following procedures to discover survival prognostic network markers (SPNs). Here we took the P-high group as an example to identify survival markers (P-intermediate and P-low groups were considered in the same manner).

We assumed a subnetwork containing *m* genes and corresponding edges extracted from the FI database^50^ (Fig. 7B). *g*_*ij*_ was labeled as the gene expression value of the i-th gene in j-th patient. First, the expression values of genes in the subnetwork were normalized to *z*_*ij*_ over all samples 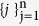, i.e. *z*_*ij*_ = (*g*_*ij*_ − *u*_*i*_). Here *u*_*i*_, *σ*_*i*_ were the mean and standard variance of *i*-th gene expression values across all samples. Second, 
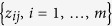
 were averaged into an activity value *x*_*j*_ for the *j*-th patient, i.e. 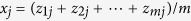. Here, the activity value 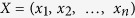 over the training set was used to predict the distant metastasis-free survival (DMFS) time *T*, i.e. 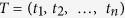. Third, we predicted the impact of activity score *X* on *T* using the Cox proportional hazard model: *H*(*t*)/*H*_0_(*t*) = exp(*β* · *X*). Score S defined as *–log(P.value)* of a chi-square test on the Cox model evaluates the impact of *X* on *T*.

Then we performed a greedy search algorithm based on score S to search the FI network database. With each gene serving as an initiating seed in one search, the search iteratively expanded initiating gene/current subnetwork by adding a gene selected from their neghboring genes, which yielded the maximal increase of score *S*. After one gene was added, we deleted a gene from current subnetwork if the score S continued to increase. At the end of this searching process, we identified a number of subnetworks called primary subnetworks (PNs) with high S scores. Considering the multiple testing, we adjusted the P-values (i.e. exp(- S score)) of PNs by controlling the false discovery rate (FDR) to obtain the adjusted S scores.

#### C. Significance test

According to step B., we identified a large group of primary subnetworks (PNs). To ensure robustness, three tests were applied on the training set to select significant PNs with favorable predictive features. The 1000 trials in the first test were based on 1000 random permutations of gene symbols (row names) of the gene expression matrix. The 1000 trials in the second test were based on 1000 random permutations of expression values of each gene across all samples, and the 1000 trials in the third test were based on 1000 random permutations of patients’ survival time (DMFS). In each trial, we conducted step B to find subnetworks together with adjusted S scores.

For each test, the 1000 trials were used as null background and 95% quantile was chosen as threshold to filter subnetworks. Through these three tests, we identified the final significant subnetwork markers, which were defined as the survival prognostic network markers (SPNs).

#### D. Survival analysis based on SPNs

*Hierarchical clustering* method (Euclidean distance) was applied to patients’ activity matrix (activity value of SPNs vs patients). After the clustering, patients were clustered into two discriminate subgroups. The hierarchical clustering was implemented by “hcluster” function in R with average linkage.

*Kaplan-Meier survival* analysis was used to study the survival of two discriminate subgroups, and the log-rank test was applied to evaluate statistical significance of survival curves. Based on the average survival, we annotated the two sub-groups as good outcome (>5 years) and poor outcome (<5 years) groups.

#### E. Validation of SPNs

Two validation strategies were used to evaluate our model. One strategy is the cross-validation^57^, which is performed for 1000 times. Taking P-high tertile as an example, we divided the P-high tertile in our breast cancer dataset (n = 652) into 10 equal subsets (10-fold cross-validation). Each of the 10 subsets of this tertile was evaluated as test set, while we trained subnetwork markers on the rest 9 subsets (training set). We used the markers identified by the training set to classify samples in the test set. After the cross-validation was complete, each sample in the P-high tertile was classified into good or poor outcome. By following the strategy in Simon, *et al*.^57^, we assigned the patients that were classified as good outcome in the 10 loops of the cross-validation together, and patients classified as poor outcome in the 10 loops of the cross-validation together. The survival curves, which were cross-validated, were constructed based on this classification, and the log-rank P-value was calculated. After implementing this procedure for 1000 times, we obtained the robust assignment of patients and the P-value for such assignment.

For independent validation, we used the test set (GSE25055) to examine the significance of SPNs identified from our breast cancer dataset. Firstly, we divided samples in the test set to three subsets (high, inter, low) by the nearest shrunken centroid classifier^25^, which was trained on the proliferation gene matrix (proliferation genes vs patient samples) of P-high, P-inter and P-low training set respectively. For a new sample, the gene expression profile was transformed into a proliferation metagene profile. The nearest shrunken centroid classifier assigned the new patient to P-high, P-inter or P-low tertile whose shrunken mean value of metagene profile was more similar to the metagene profile of the new sample. Then, for each subset, we applied the corresponding SPN (P-high/P-inter/P-low SPN) markers to divide it into good outcome group and poor outcome group.

#### F. Bio-function analysis

*Enrichment in biological processes and functional pathways* the biological process (BP) sets and KEGG pathway sets were downloaded from MSigDB database in GSEA. We performed an enrichment analysis of our SPNs by comparing genes in each SPN to the biological processes and pathways. Fisher’s exact test was conducted to identify enriched biological process and pathway categories, and to suggest the most important biological functions associated with our SPNs. One biological process or pathway is “enriched” if the fisher’s test P-value corrected using Bonferroni adjustments is less than 0.05, where the adjusted P-value evaluates the significance of enrichment for certain category in our SPNs. R software was used to visualize the BP and KEGG sets enrichment.

#### G. Comparison with other algorithm

We analyzed the comparative performances of subnetwork signatures identified by our method with that of the Cox-based Ridge regression method^22^,^23^ and the CRANE algorithm^24^. The Cox-based Ridge regression method is the Cox regression model that is regularized with L2 penalty in Ridge regression. The CRANE algorithm identifies subnetworks (together with subnetwork states that is a specific combination of quantitated mRNA expression levels of genes in a subnetwork) that are coordinately dysregulated in tumorigenic and metastatic samples. CRANE developes a combinatorial formula of coordinate dysregulation of a subnetwork in terms of mutual information between the subnetwork activity and phenotype. Then the combinatorial fomula is decomposed into individual terms, which measures the information that individual subnetwork state provided on phenotype (e.g. metastasis). With the statistical property of individual subnetwork state, they proposed a bottom-up searching algorithm that can effectively prune out the subnetwork space to identify informative subnetworks (Details in 24). The informative subnetworks identified by CRANE algorithm were then compared with the performance of our SPNs.

Three criteria, i.e. accuracy, precision and recall, were used to measure the classification performance. According to the definitions^58^ and the concepts in this study, we define a true positive as a metastatic sample that is correctly predicted as a metastatic sample, while a false positive is a non-metastatic sample that is incorrectly predicted as metastatic. A true negative is defined as a non-metastatic sample that is correctly predicted as non-metastatic, while a false negative is a metastatic sample that is incorrectly predicted as non-metastatic. Then the three performance criteria are defined as: Accuracy = (# true positives + # true negatives)/(# false positives + # false negatives); Precision = # true positives/(# true positives + # false positives); Recall = # true positives/(# true positives + # false negatives), where # refers to number of samples. As defined, the accuracy quantifies thse proportion of true results (including true positives and true negatives) among all samples. The precision quantifies the proportion of true positives among all samples predicted as metastatic, while recall quantifies the proportion of true positives among all metastatic samples.

## Additional Information

**How to cite this article**: Song, Q. *et al*. Systems biology approach to studying proliferation- dependent prognostic subnetworks in breast cancer. *Sci. Rep.* **5**, 12981; doi: 10.1038/srep12981 (2015).

## Supplementary Material

Supplementary Information
