# Supplementary material for: Systems biology approach to studying proliferation-dependent prognostic subnetworks in breast cancer
Source: Sci Rep. 2015 Aug 10;5:12981. doi: 10.1038/srep12981 (PMC4530341; doi:10.1038/srep12981)
Supplement: Supplementary Information [file srep12981-s1.pdf]

Systems biology approach to studying proliferation-dependent prognostic subnetworks in breast cancer

Qianqian Song[1,2], Hongyan Wang[1], Jiguang Bao[2], Ashok K. Pullikuth[3], King C. Li[1], Lance D. Miller[3] and Xiaobo Zhou[1,3,*]

[1]Division of Radiology, Wake Forest School of Medicine, Winston-Salem, NC 27157, USA

[2]School of Mathematical Sciences, Beijing Normal University, Beijing, 100875, P R China

[3]Department of Cancer Biology, Wake Forest School of Medicine, Winston-Salem, NC, 27157, USA

*Corresponding author

Email addresses:

Q.S.: wasqqdyx@gmail.com

H.W.: jojowang.business@gmail.com

J.B.: jgbao@bnu.edu.cn

A.K.P.: apulliku@wakehealth.edu

K.C.L.: kingli@wakehealth.edu

L.D.M.: ldmiller@wakehealth.edu

X.Z.: xizhou@wakehealth.edu

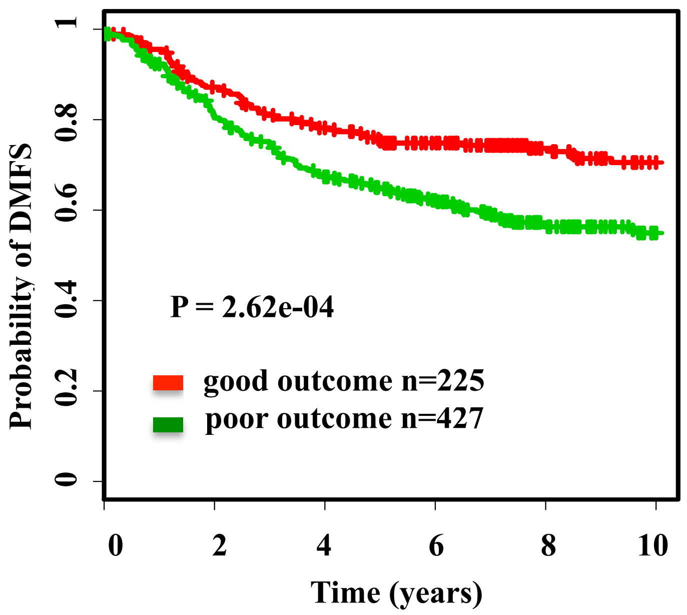

Figure S1. Cross-validated Kaplan-Meier survival curves of P-high tertile in our BR dataset.

The figure presented the result of cross-validation on our BR dataset. The red color represented good outcome patients (low-risk), while the green color represented poor outcome patients (high-risk). The significant P-value declared the robustness of our model.

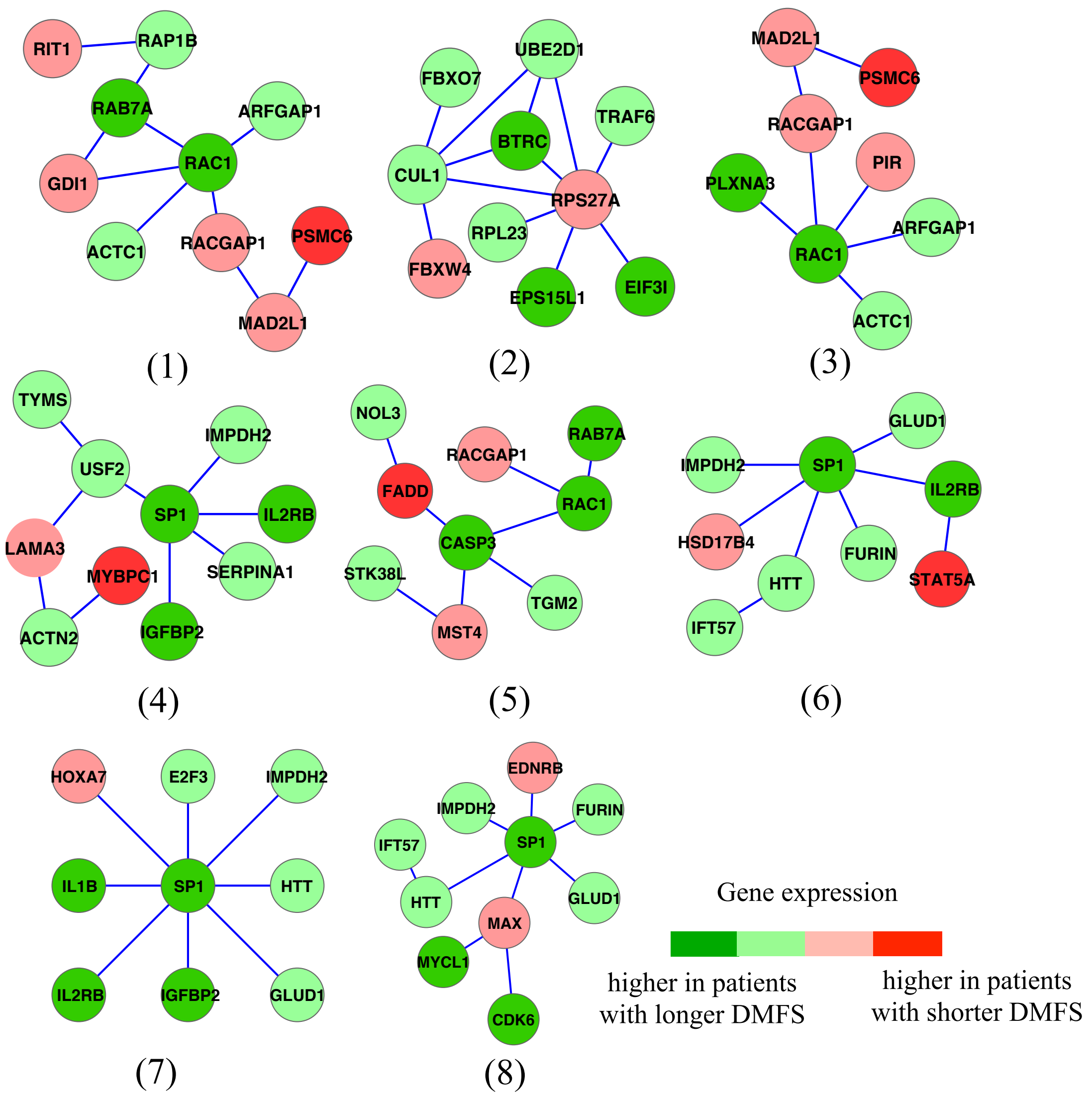

Figure S2. SPNs in the P-inter group, i.e. P-inter SPNs.

There are 8 SPNs discovered in the inter proliferation (P-inter) group. Genes are color coded (i.e. red/green: genes that are overexpressed/underexpressed in patients with shorter DMFS). In the color bar, deep green means that log2(fold change)<0, ligh green or light red represents that 0<fold change<1， while deep red means that log2(fold change)>0. Details about P-inter SPNs can be found in the Results section.

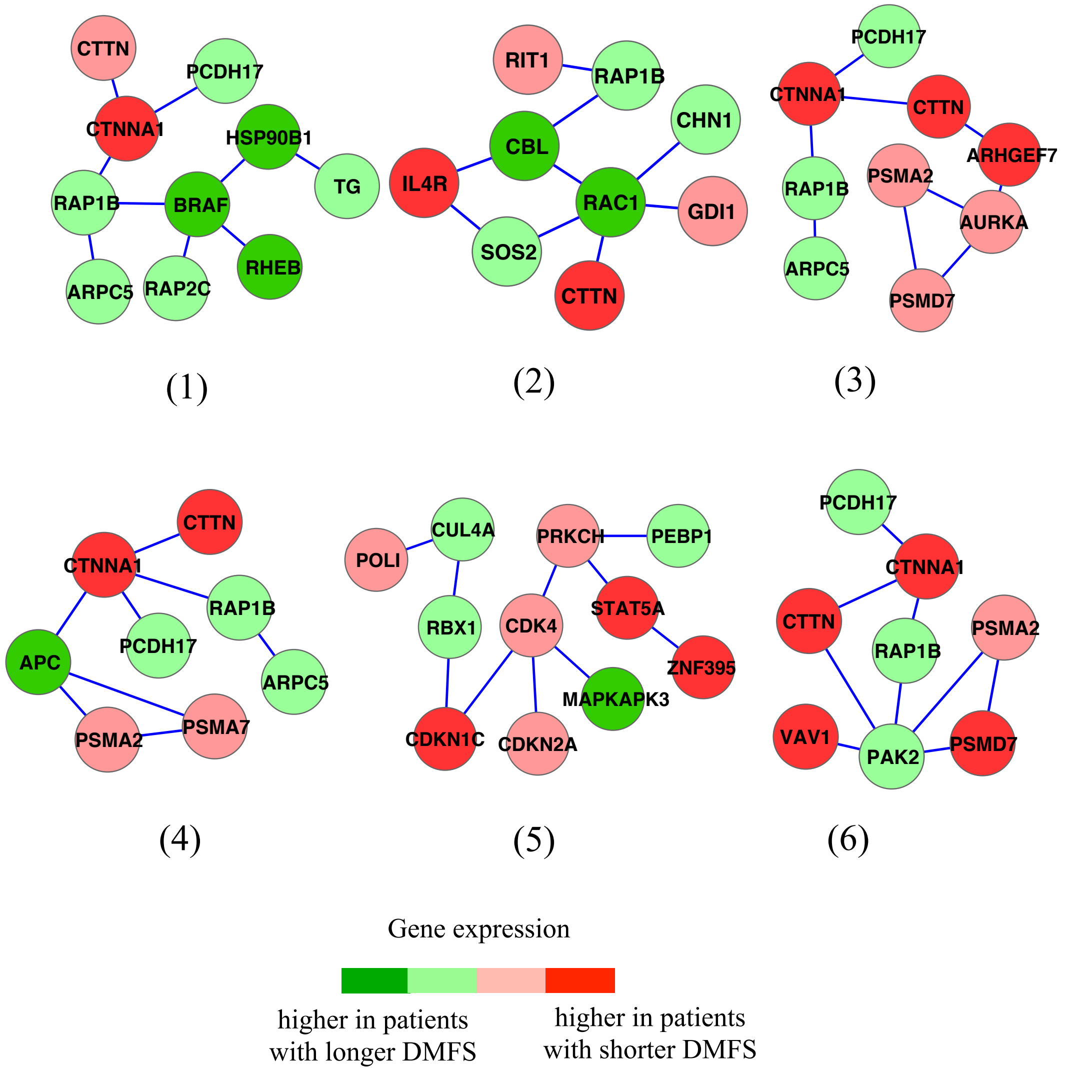

Figure S3. SPNs in the P-low group, i.e. P-low SPNs.

There are 6 SPNs discovered in the low proliferation (P-low) group. Genes are color coded (i.e. red/green: genes that are overexpressed/underexpressed in patients with shorter DMFS). In the color bar, deep green means that log2(fold change)<0, ligh green or light red represents that 0<fold change<1, while deep red means that log2(fold change)>0. Details about P-low SPNs can be found in the Results section.

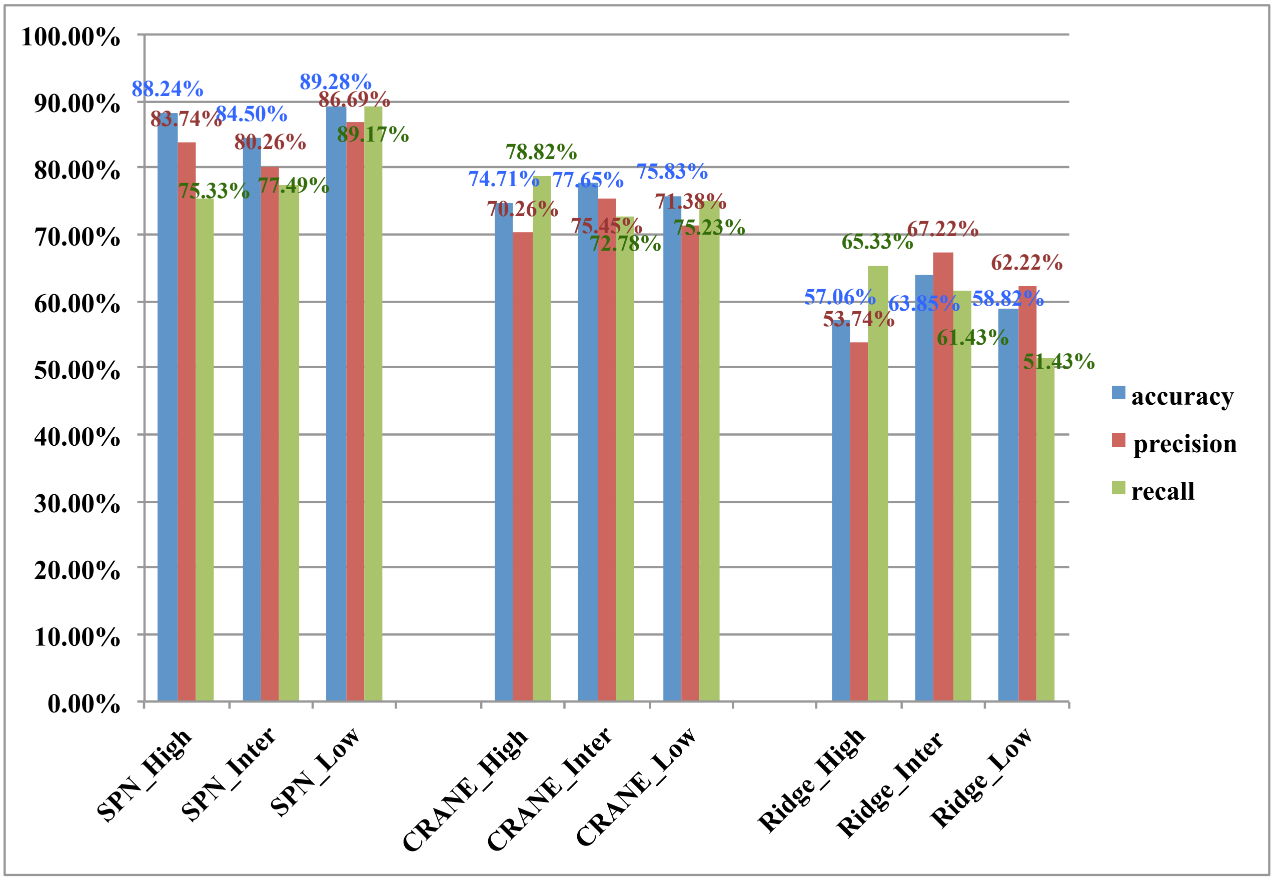

Figure S4. Classification performance of makers identified by different methods in predicting breast cancer metastasis.

We calculated the accuracy, precision and recall of correct classification into metastatic-/non-metastatic patient groups based on our identified SPNs. The color of number corresponds to the color of bar, for example, in the P-high test set, the accuracy, precision and recall are 88.24%, 83.74%, 75.33%, respectively. The classification accuracy, precision and recall achieved by different methods (the Ridge-based Cox model and CRANE method) were also reported in the figure.

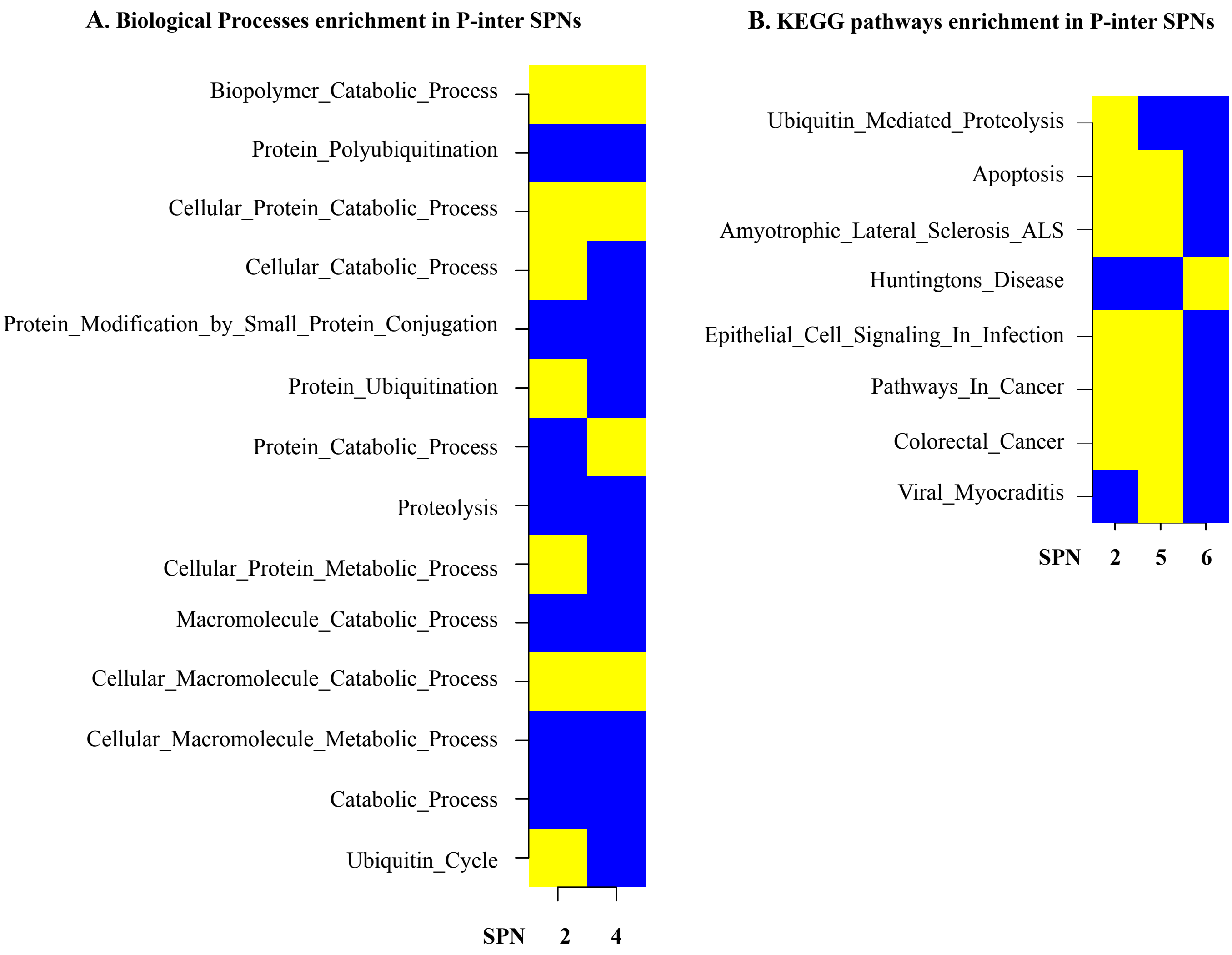

Figure S5. Enrichment analysis of P-inter SPNs in BP sets and KEGG pathway sets. Subfigure A. showed the enrichment of P-inter SPNs in biological process (BP) sets, while subfigure B. was the enrichment of P-inter SPNs in KEGG pathway sets. Enriched biological process or pathway (i.e. enrichment) was indicated by yellow, whereas non-enrichment was indicated by blue.

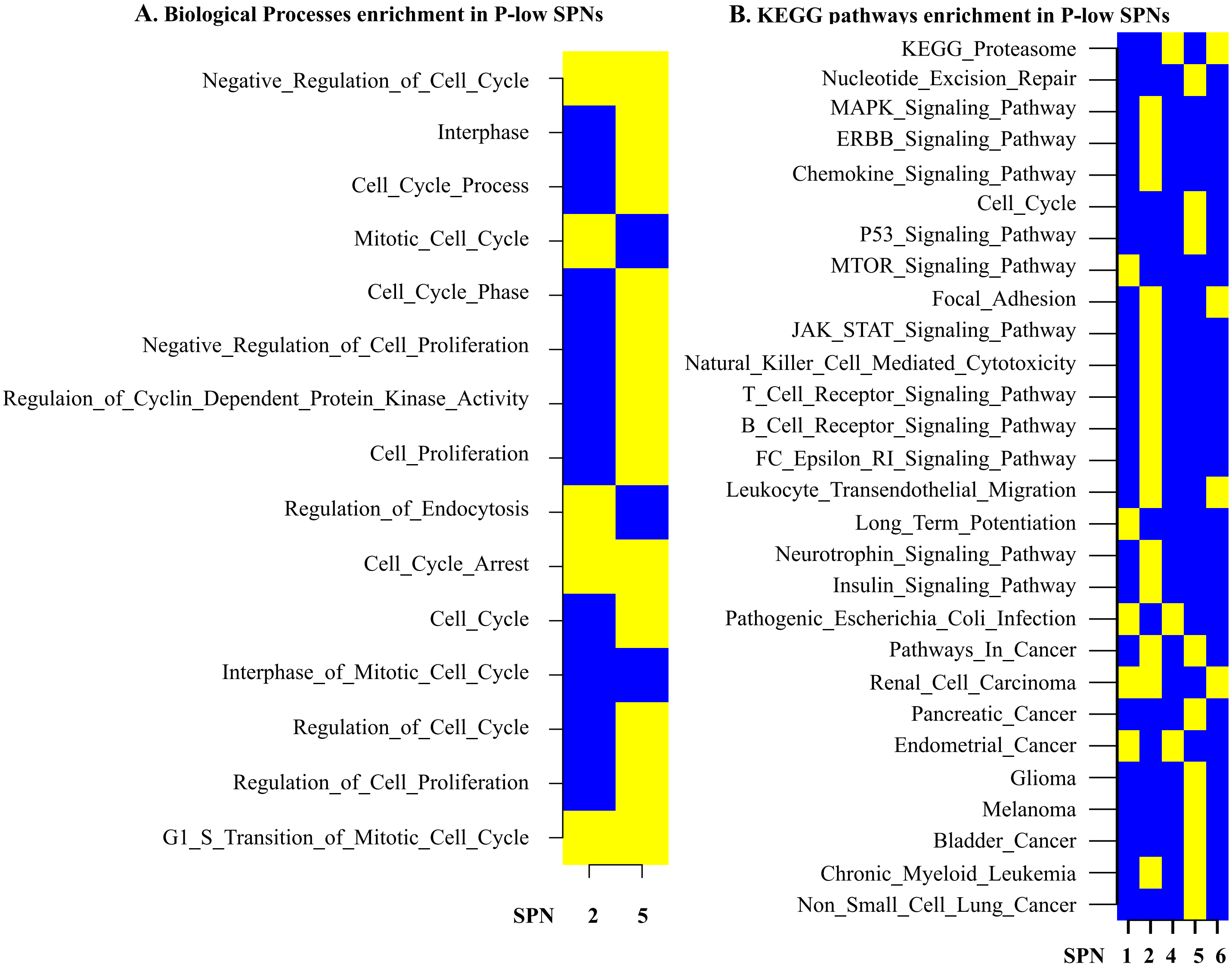

Figure S6. Enrichment analysis of P-low SPNs in BP sets and KEGG pathway sets. Subfigure A. showed the enrichment of P-low SPNs in biological process (BP) sets, while subfigure B. was the enrichment of P-low SPNs in KEGG pathway sets. Enriched biological process or pathway (i.e. enrichment) was indicated by yellow, whereas non-enrichment was indicated by blue.

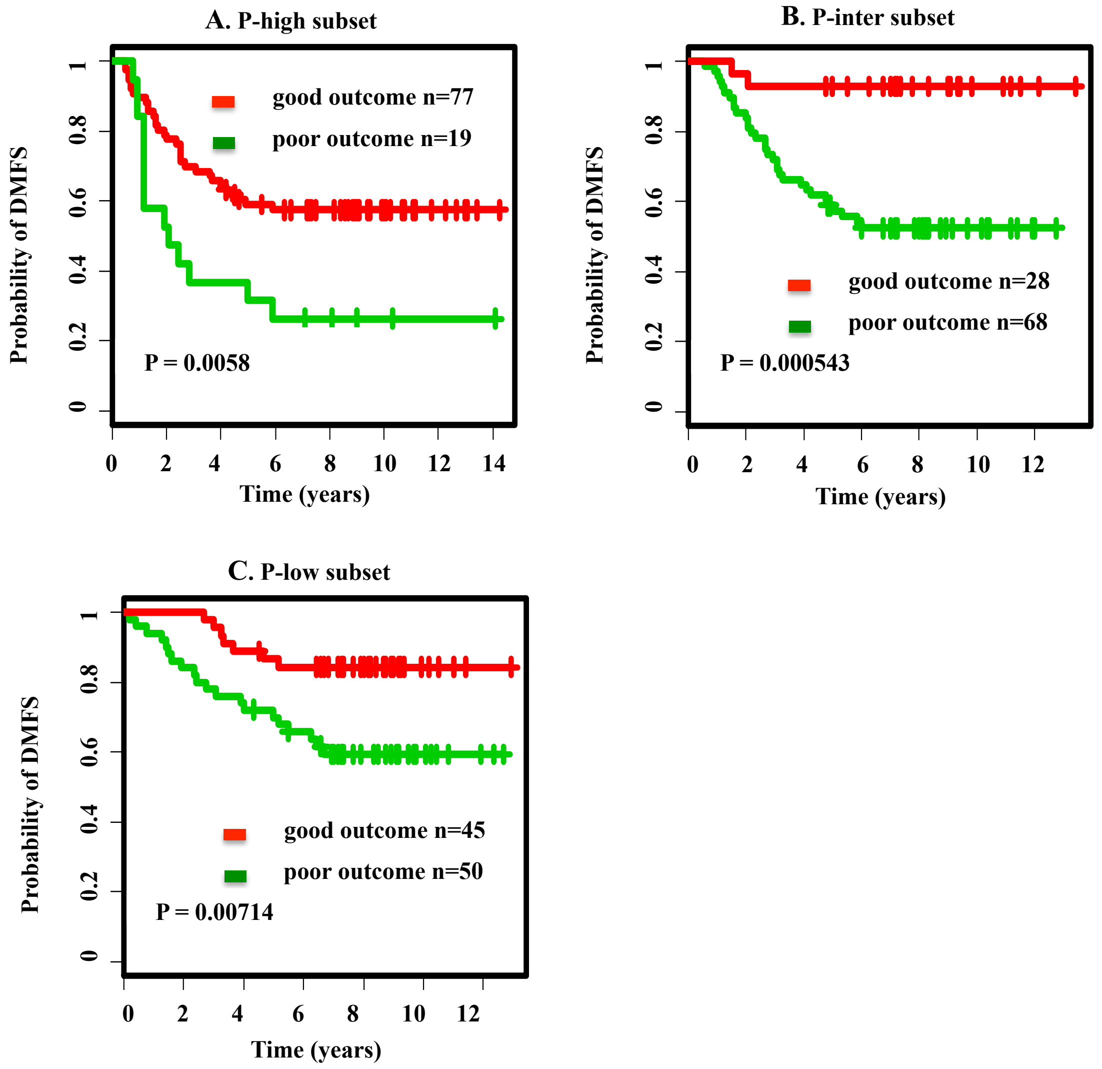

Figure S7. Survival analysis of the dataset used in Wang et al.

A. shows the survival curves (with log-rank P-value) of P-high subset in Wang et al.'s dataset. **B.** shows the survival curves of P-inter subset in Wang et al.'s dataset. **C.** shows the survival curves of P-low subset in Wang et al.'s dataset.

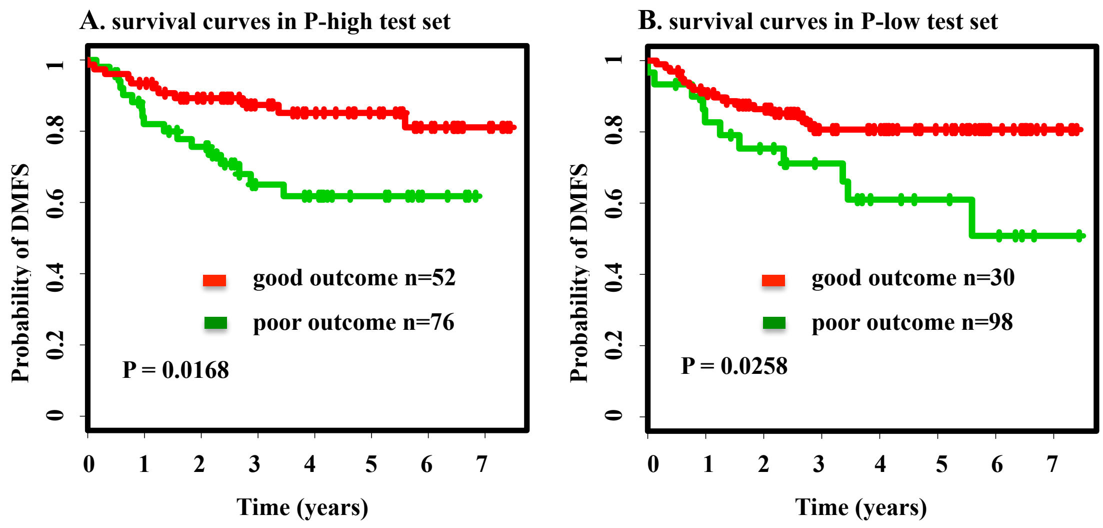

Figure S8. Survival curves of two proliferation groups (P-high group, P-low group) in test set.

A. shows the survival analysis of P-high group in test set. **B**. shows the survival analysis of P-low group in test set.

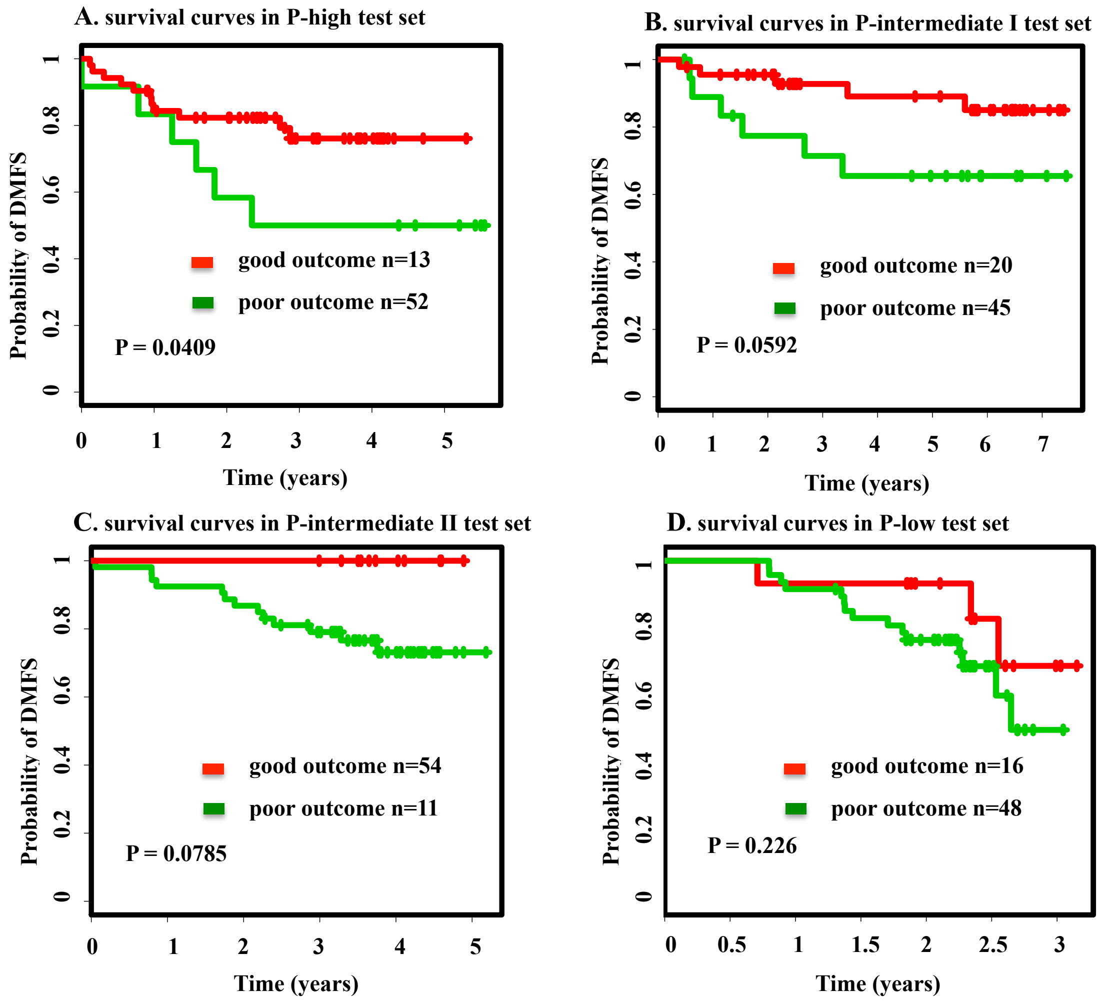

Figure S9. Survival curves of four proliferation groups (P-high, P-intermediate I, P-intermediate II , P-low group) in test set.

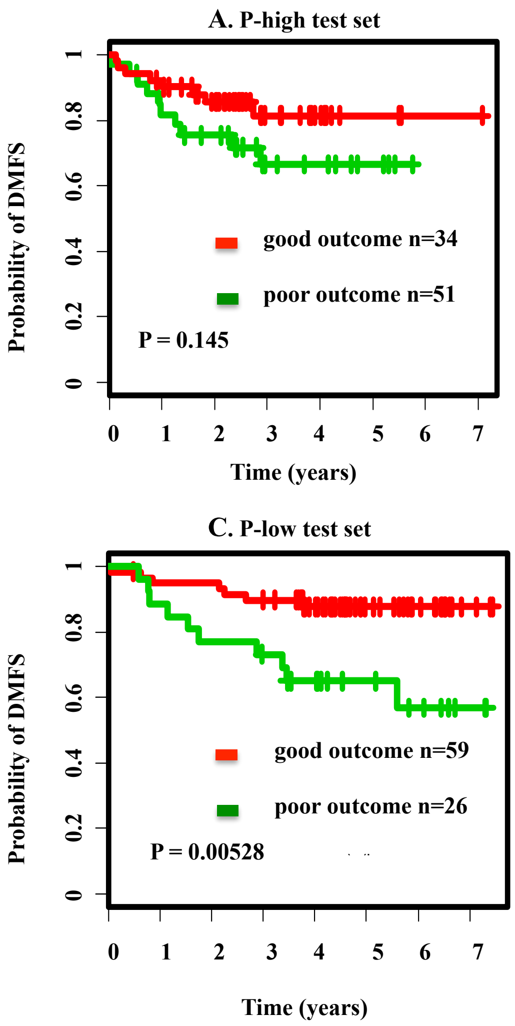

Figure S10. Survival analysis of three proliferation tertiles with weights in SPNs.

A. shows the survival curves of P-high test set based on the weighted P-high SPNs. **B**. shows the survival curves and log-rank P-value of P-inter test set based on the weighted P-inter SPNs. **C**. shows the survival curves of P-low test set based on the weighted P-low SPNs.

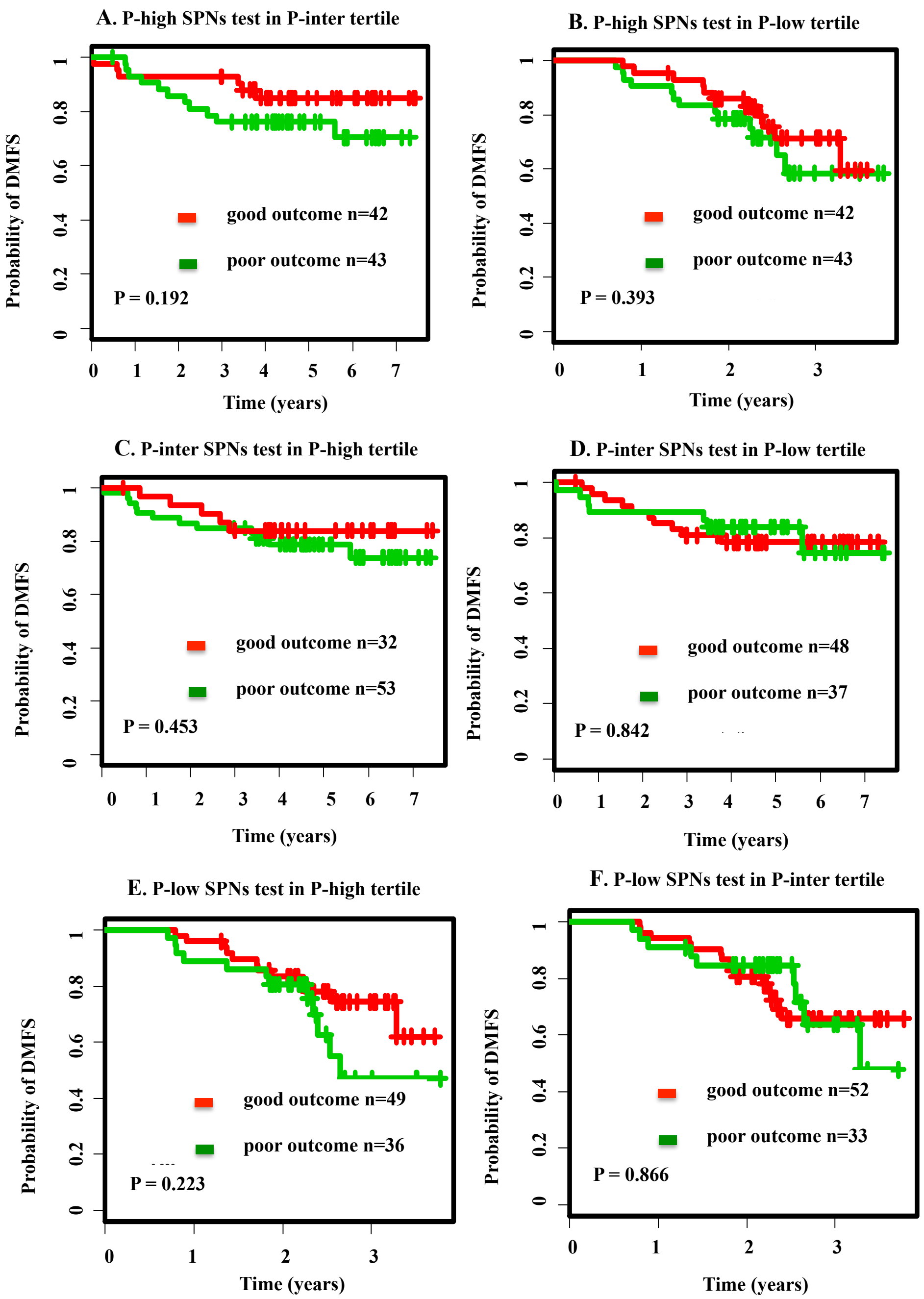

Figure S11. Test in different proliferation tertiles by SPNs.

A. shows the survival curves of P-inter group based on P-high SPNs. **B**. shows the survival curves of P-low group based on P-high SPNs. **C**. shows the survival curves of P-high group based on P-inter SPNs. **D**. shows the survival curves of P-low group based on

P-inter SPNs. **E**. shows the survival curves of P-high group based on P-low SPNs. **F**. shows the survival curves of P-inter group based on P-low SPNs.

Supplementary Table S1. Adjusted P-values of P-high, P-inter and P-low SPNs.

adjust P-value of SPNs	#1	#2	#3	#4	#5	#6	#7	#8
P-high	1.22E-16	1.11E-16	2.22E-16	5.11E-16	1.45E-16	1.11E-16	1.67E-16	5.55E-16
P-inter	1.11E-16	6.02E-15	1.11E-15	1.12E-16	2.45E-16	1.39E-15	3.77E-15	2.22E-16
P-low	1.67E-15	1.33E-15	1.23E-15	1.22E-15	9.99E-16	3.33E-16		

Supplementary Table S2. Clinical and pathological characteristics of test set.

Characteristics	test set* (n=255)
Age,years	
<=40	46 (18.43%)
41-55	128 (50.2%)
56-70	74 (29%)
>70	7 (2.37%)
T stage	
T0	2 (0.78%)
T1	19 (7.45%)
T2	133 (52.16%)
T3/4	101 (39.61%)
Grade	
Poor	121 (47.45%)
Moderate	98 (38.43%)
Good	13 (5.1%)
Unkown	23 (9.02%)
ER status	
Positive	148 (58.04%)
Negative	107 (41.96%)
Subtype	
Normal	24 (9.41%)
LumA	81 (31.77%)
LumB	34 (13.3%)
Her2	19 (7.45%)
Basal	97 (38.04%)
Metastasis within 5 years	
Yes	59 (23.14%)
No	196 (76.86%)

* samples in test set are extracted from GSE25055

Supplementary Table S3. Affymetrix probe sets and gene names that comprise the proliferation metagene.

Proliferation Metagene		
**Probe Set ID**	**Gene Symbol**	**Gene Name**
201291_s_at	TOP2A	Topoisomerase (DNA) II alpha 170kDa
201292_at	TOP2A	Topoisomerase (DNA) II alpha 170kDa
201890_at	RRM2	Ribonucleotide reductase M2
202095_s_at	BIRC5	Baculoviral IAP repeat-containing 5
202503_s_at	KIAA0101	KIAA0101
202580_x_at	FOXM1	Forkhead box M1
202589_at	TYMS	Thymidylate synthetase
202705_at	CCNB2	Cyclin B2
202870_s_at	CDC20	Cell division cycle 20 homolog (S. cerevisiae)
202954_at	UBE2C	Ubiquitin-conjugating enzyme E2C
203213_at	CDK1	Cyclin-dependent kinase 1
203214_x_at	CDK1	Cyclin-dependent kinase 1
203362_s_at	MAD2L1	MAD2 mitotic arrest deficient-like 1 (yeast)
203554_x_at	PTTG1	Pituitary tumor-transforming 1
203755_at	BUB1B	Budding uninhibited by benzimidazoles 1 homolog beta (yeast)
203764_at	DLGAP5	Discs, large (Drosophila) homolog-associated protein 5
204026_s_at	ZWINT	ZW10 interactor
204033_at	TRIP13	Thyroid hormone receptor interactor 13
204092_s_at	AURKA	Aurora kinase A
208079_s_at	AURKA	Aurora kinase A
204162_at	NDC80	NDC80 homolog, kinetochore complex component (S. cerevisiae)
204170_s_at	CKS2	CDC28 protein kinase regulatory subunit 2
204444_at	KIF11	Kinesin family member 11
204641_at	NEK2	NIMA (never in mitosis gene a)-related kinase 2
204822_at	TTK	TTK protein kinase
204825_at	MELK	Maternal embryonic leucine zipper kinase
204962_s_at	CENPA	Centromere protein A
205034_at	CCNE2	cyclin E2
205046_at	CENPE	Centromere protein E, 312kDa
206102_at	GINS1	GINS complex subunit 1 (Psf1 homolog)
206364_at	KIF14	Kinesin family member 14
207828_s_at	CENPF	Centromere protein F, 350/400kDa (mitosin)
209172_s_at	CENPF	Centromere protein F, 350/400kDa (mitosin)
209408_at	KIF2C	Kinesin family member 2C
209642_at	BUB1	Budding uninhibited by benzimidazoles 1 homolog (yeast)
209714_s_at	CDKN3	Cyclin-dependent kinase inhibitor 3
209773_s_at	RRM2	Ribonucleotide reductase M2
210052_s_at	TPX2	TPX2, microtubule-associated, homolog (Xenopus laevis)
210559_s_at	CDK1	Cyclin-dependent kinase 1
212022_s_at	MKI67	Antigen identified by monoclonal antibody Ki-67
212949_at	NCAPH	Non-SMC condensin I complex, subunit H
213226_at	CCNA2	Cyclin A2
214710_s_at	CCNB1	Cyclin B1
218009_s_at	PRC1	Protein regulator of cytokinesis 1
218039_at	NUSAP1	Nucleolar and spindle associated protein 1
218355_at	KIF4A	Kinesin family member 4A
218542_at	CEP55	Centrosomal protein 55kDa
218585_s_at	DTL	Denticleless homolog (Drosophila)
218662_s_at	NCAPG	Non-SMC condensin I complex, subunit G
218663_at	NCAPG	Non-SMC condensin I complex, subunit G
218726_at	HJURP	Holliday junction recognition protein
218755_at	KIF20A	Kinesin family member 20A
218883_s_at	MLF1IP	MLF1 interacting protein
219148_at	PBK	PDZ binding kinase
219306_at	KIF15	Kinesin family member 15
219918_s_at	ASPM	asp (abnormal spindle) homolog, microcephaly associated (Drosophila)
220651_s_at	MCM10	Minichromosome maintenance complex component 10
221436_s_at	CDCA3	Cell division cycle associated 3
221520_s_at	CDCA8	Cell division cycle associated 8
222039_at	KIF18B	Kinesin family member 18B
222077_s_at	RACGAP1	Rac GTPase activating protein 1
204562_at	IRF4	Interferon regulatory factor 4

Supplementary Table S4. Overlap of genes among the SPNs, Chuang et.al, CRANE and the Cox-based Ridge regression method.

	SPN								CRANE											Ridge
Gene Symbol	1	2	3	4	5	6	7	8	1	2	3	4	5	6	7	8	9	10	11	1
**GNG11**	■																			
**TUBB6**	■	■																		
COBRA1			■																	
**SMAD2**				■			■					■								
**CALM1**				■	■		■													■
**IGFBP7**						■														
**DCTN2**							■													
**HSPA1A**	■	■		■	■	■		■												
**TBCA**		■		■	■	■		■												
**MNAT1**			■							■										
**ASPM**				■																
CBX3					■									■						
POLR2H							■													
**HSP90AA1**	■	■		■	■	■		■												■
**TBCE**	■							■												
**TUBB2C**	■	■		■	■	■		■												■
STK3			■																	
**CALD1**				■					■											
**RDBP**							■													
**CDK7**			■				■													
CCR9	■																			
**SFN**			■				■													
NPM1		■															■			
**CTTN**			■																	■
**IGF1R**						■					■									■
**TUBA1A**								■												
**TAX1BP1**		■																		
LAMA3			■																	
**XPO1**					■															
**VEGFA**						■				■										
STUB1	■																			
KTN1		■													■					
THBS3			■																	
GNG10							■													
UBE2D2	■																			
**SPP1**			■																	
DNAJA3				■																
**RAP1GAP**							■									■				
RNF41	■									■										

**ITGA3**
GLI3
**CD151**
FBXW11
CHST2
NUDT9
PRKCZ
FEZ2
VAV1
IL6ST
KRT10
NLRP1
MCM7
YWHAQ
MAGOH
CR1
PCGF2
SMARCA5
GRB14
AKAP8
ASF1A
RAD21
TRAF6
NFKB1
C3
PDGFRA
CHEK2
SKAP1
CREB3L2
M6PR
SP140L
TAF9
PSMB10
TXNIP
TAPBPL
ARHGEF18
LPIN1
DTNB
NDUFS6
BTF3
IP6K1
CD72
PTGER4
MSN

BCL11A			
CCDC88A			
TNNT1			
CD37			
QKI			
TCEAL4			
RARRES1			
